# Increased Fitness of Rice Plants to Abiotic Stress Via Habitat Adapted Symbiosis: A Strategy for Mitigating Impacts of Climate Change

**DOI:** 10.1371/journal.pone.0014823

**Published:** 2011-07-05

**Authors:** Regina S. Redman, Yong Ok Kim, Claire J. D. A. Woodward, Chris Greer, Luis Espino, Sharon L. Doty, Rusty J. Rodriguez

**Affiliations:** 1 Western Fisheries Research Center, United States Geological Survey, Seattle, Washington, United States of America; 2 College of Forest Resources, University of Washington, Seattle, Washington, United States of America; 3 Biology Department, University of Washington, Seattle, Washington, United States of America; 4 University of California Cooperative Extension, Yuba City, California, United States of America; 5 University of California Cooperative Extension, Colusa, California, United States of America; Cairo University, Egypt

## Abstract

Climate change and catastrophic events have contributed to rice shortages in several regions due to decreased water availability and soil salinization. Although not adapted to salt or drought stress, two commercial rice varieties achieved tolerance to these stresses by colonizing them with Class 2 fungal endophytes isolated from plants growing across moisture and salinity gradients.

Plant growth and development, water usage, ROS sensitivity and osmolytes were measured with and without stress under controlled conditions.

The endophytes conferred salt, drought and cold tolerance to growth chamber and greenhouse grown plants. Endophytes reduced water consumption by 20–30% and increased growth rate, reproductive yield, and biomass of greenhouse grown plants. In the absence of stress, there was no apparent cost of the endophytes to plants, however, endophyte colonization decreased from 100% at planting to 65% compared to greenhouse plants grown under continual stress (maintained 100% colonization).

These findings indicate that rice plants can exhibit enhanced stress tolerance via symbiosis with Class 2 endophytes, and suggest that symbiotic technology may be useful in mitigating impacts of climate change on other crops and expanding agricultural production onto marginal lands.

## Introduction

The geographic distribution pattern of plants is thought to be based on climatic and edaphic heterogeneity that occurs across complex habitats [1], [2], [3]. All plants express some level of phenotypic plasticity [4] enabling them to grow in diverse habitats and across environmental gradients [5], [6], [7], [8], [9]. Phenotypic plasticity is defined as the production of multiple phenotypes from a single genotype, depending on environmental conditions and is considered adaptive if it is maintained by natural selection [4], [9], [10].

Plant adaptation to high stress habitats likely involves a combination of phenotypic plasticity and genetic adaptation, and is thought to involve processes exclusive to the plant genome [11], [12], [13], [14]. However, the mechanisms responsible for adaptation to high stress habitats are poorly defined. For example, all plants are known to perceive, transmit signals and respond to abiotic stresses such as drought, heat, and salinity [15], [16]. Yet, few species are able to colonize high stress habitats which typically have decreased levels of plant abundance compared to adjacent low stress habitats [17], [18]. Although there are numerous reports on the genetic, molecular and physiological bases of how plants respond to stress, the nature of plant adaptation to high stress habitats remains unresolved [19], [20], [21]. However, most ecological studies fail to consider the fact that all plants in natural ecosystems are thought to be symbiotic with fungal endophytes and these endophytes can have profound effects on plant stress tolerance and fitness [22], [23]. For example, fungal endophytes can confer fitness benefits to plants including increased root and shoot biomass, increased yield, tolerance to abiotic stresses such as heat, salt, and drought, and to biotic stresses such as pathogens and herbivores [24], [25], [26], [27], [28], [29], [30], [31]. One group of fungal endophytes [Class 2; [32]] confer habitat-specific stress tolerance to plants through a process defined as Habitat Adapted Symbiosis [33]. Remarkably, Class 2 endophytes, are capable of colonizing and conferring habitat-specific stress tolerance to monocot and eudicot plants which may suggest that the symbiotic communication responsible for stress tolerance may predate the divergence of these lineages (est. 145–230 mya) [34], [35], [36].

During the last several decades, there have been major climatic events that decreased agricultural productivity of rice (one of the four major food crops) at locations around the world. For example, in 2004, an earthquake generated tidal wave flooded Indonesia [37], and in 2008, a cyclone resulted in a tidal surge that flooded southern Burma. Both of these events resulted in inundation of productive agricultural lands with salt water that decreased or eliminated production of rice for one or more years. During the last 40 years of climate change, increased minimum air temperatures during growing seasons have resulted in a substantial decrease in rice yields in China and the Philippines and are predicted to continue [38], [39]. Collectively, these events along with increasing world-population, have contributed to shortages and increased prices of rice exacerbating hunger and famine issues globally. Climate change has also begun to alter the phenology of currently used commercial rice varieties making predictions about rice availability and abundance less reliable. Although it may be possible to compensate for some impacts of phenology shifts by incorporating earlier season varieties into agricultural practices [40], the adaptive capabilities of rice will ultimately determine the severity of climate change on annual crop yields. However, the adaptive potential of most plants, including rice, is not well characterized. Here we report that the ability of rice to rapidly exhibit stress tolerance is dependent on associations with Class 2 fungal endophytes. The influence of three endophytes on the ability of rice to tolerate low temperatures, high salinity and desiccation was tested. In addition, the influence of the endophytes on growth, development and water usage was assessed under greenhouse and laboratory conditions. The potential for using symbiotic technologies to mitigate the impacts of climate change is discussed.

## Results

### Growth Chamber/Greenhouse Studies

#### Fitness benefits (growth, development and yield)

**Figure 1:**
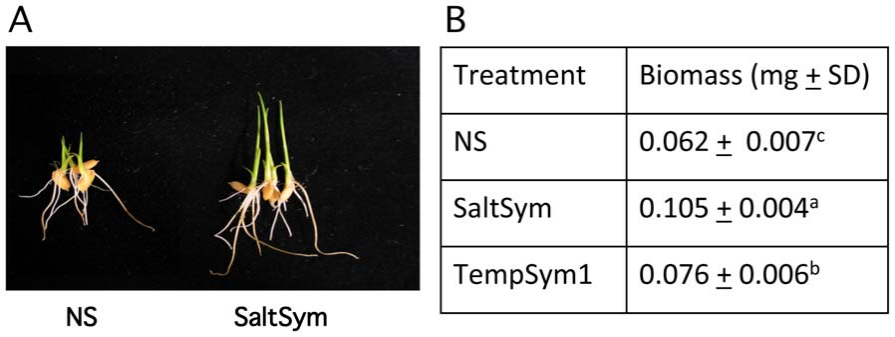
Effects of symbiosis on growth response on young seedlings in the absence of stress. The number of plants/treatment are indicated (N = XX) below. Statistical analysis was performed using Duncan's multiple-range test. Values with the same letters are not significantly different. **A**) Representative photo of three day old rice seedlings (N = 30 total) that were nonsymbiotic (NS) or symbiotic (S) with SaltSym. **B**) Growth of rice seedlings were measured by assessing the dry biomass (mg) of (N = 10/rep) three day old rice seedling that were NS or S with the SaltSym or TempSym1. Each assay was repeated three times. Statistical analysis indicated that S plants were statistically larger (SD≤0.06; P≤0.0002) than NS plants.

**Figure 2:**
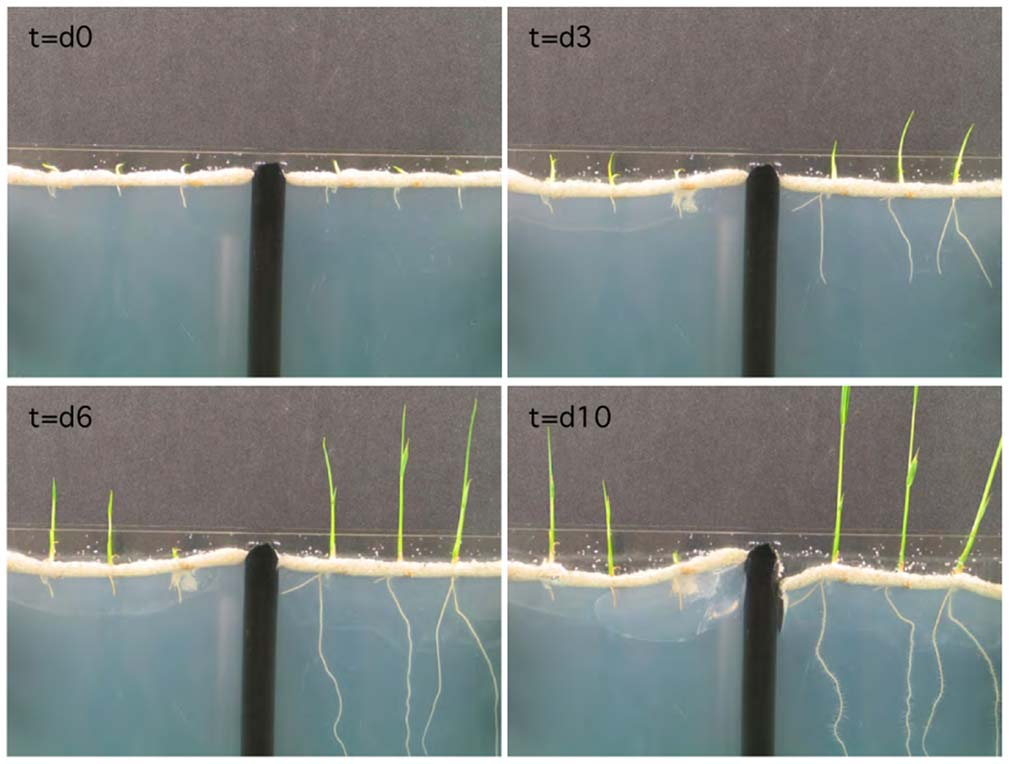
Representative time-lapse photos of rice growth and development. NS (left panel) and S (SaltSym; right panel) rice seedlings (N = 3) were photographed every 20 min over a ten-day period. Sterilized, imbibed rice seeds were inoculated with fungal spore solutions (S) or mock-inoculated (NS) for 48 hours on water agar plates until seed germination occurred. Three S and NS seedlings were randomly chosen and imbedded into the silica sand layer on top of the vertical plant growth apparatus, and the time-lapse photography initiated (identified as time point t = 0). Zero, three, six and ten day time points are represented by t = 0, t = d3, t = d6, and t = d10, respectively.

Both *Fusarium culmorum* isolate FcRed1 (SaltSym) and *Curvularia protuberata* isolate Cp4666D (TempSym1) (Table 1) significantly increased the growth and development of seedlings in the absence of stress compared to nonsymbioitic plants (Fig.1 & 2). Growth responses were evident less than 24 hr post inoculation and symbiotic seedlings averaged between 20 to 68% greater biomass than nonsymbiotic plants after 3 days of growth depending on the symbiotic association.

**Table 1:** Isolation of Fungal Endophytes and Predicted Symbiotic Benefits.

Fungal Species (Isolate)^a^	Acronym^b^	Habitat of Origin^c^	Habitat Specific Stress^d^
*Fusarium culmorum* (FcRed1)	SaltSym	Costal Beach	Salt & Drought
*Curvularia protuberata* (Cp4666D)	TempSym1	Geothermal soil-H	High Temp. & Drought
*Curvularia protuberata* (CpYNP5C)	TempSym2	Geothermal soil-L	Low Temp. & Drought

a Fungal species were isolated from plants growing in native habitats.

b Fungal isolates referred to in the text as SaltSym or TempSym1&2 and confer salt and temperature tolerance, respectively. Sym  =  Symbiont.

c Native habitat in which fungal isolates were originally obtained. Coastal beaches were located in the San Juan Islands, Washington and the geothermal soils were in Yellowstone National Park, Wyoming. Seasonal temperatures ranged from 20–55°C (Geothermal soil-H) and 5–30°C (Geothermal soil-L).

d The identifiable stress in native habitats. The predicted stress tolerance fungal endophytes will confer to rice plants.

**Figure 3:**
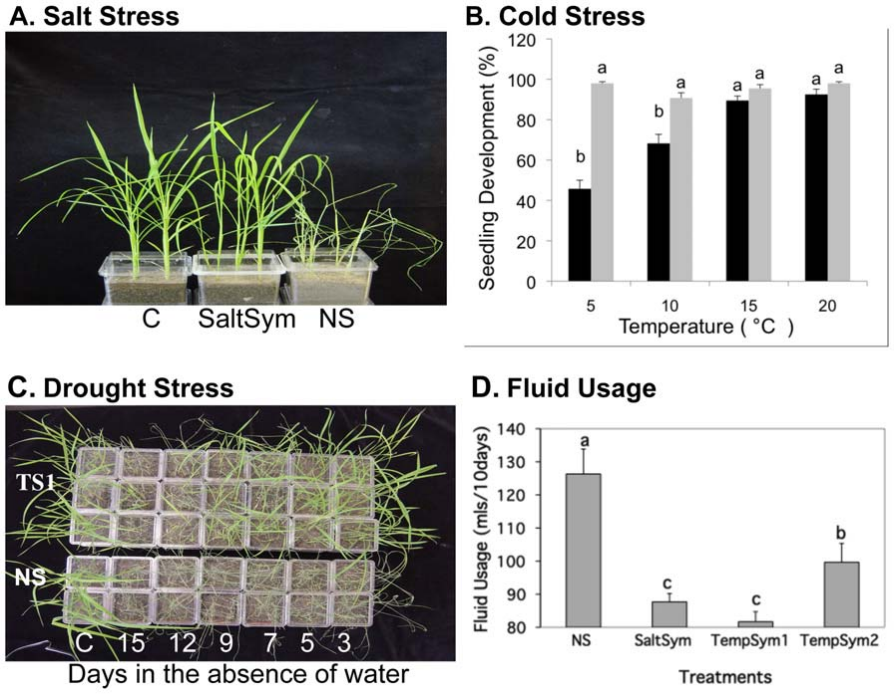
Effects of salt, cold and drought stress and water usage in S and NS rice plants under laboratory conditions. The number of plants or seedlings/treatment are indicated by (N = XX), and the % survival and health of surviving plants is indicated in parentheses after each treatment (Fig. 3A &C). Plant health was based on comparison to NS controls and rated from 1 to 5 (1 = dead, 2 = severely wilted and chlorotic, 3 = wilted +/− chlorosis, 4 = slightly wilted, 5 = healthy w/o lesions or wilting). All assays described from left to right and images are representative of all plants/treatment. **A**) Rice plants (N = 60), no stress controls (labeled "C") representative of both S and NS plants (100%, 5), S with SaltSym (100%, 5), or NS (0%, 1) exposed to 300 mM NaCl for 21 days. While all plants bent over with age, unstressed controls and salt exposed S plants remained hydrated while the NS plants wilted. **B**) The % rice seedling development at 5–20°C of NS (black bars) and TempSym1 colonized (grey bars) treatments were assessed. After the initial sterilization and imbibing process (see Materials and Methods), seeds (N = 20) were placed on agar water media plates, and +/− inoculated with the fungal symbiont, and % seedling development assessed after ten days. Statistical analysis (Duncan's multiple-range test; SE≤4.48; P≤0.001) indicated the % seedling development was significantly higher at 5°C and 10°C in S treatments but not at 15°C and 20°C. **C**) Representative photo of five week old rice plants (N = 140–210) that are NS or S with TempSym1 (TS1). From Left to right: control plants (C) were kept hydrated and healthy (100%, 5) for both treatments. The remaining plants were not watered for 15,12, 9,7, 5, and 3 days. Post drought stress, plants were re-hydrated for 2 days and viability assessed. After 3 days, NS plants began to show the effects of drought (70%, 2; 25%, 1) and after>5 days, NS plants succumbed to the stress (>90%, 1). In contrast, S plants did not show the effects of drought until after 12 days (70%, 1; 30%, 2). Moreover, S plants remained green and robust in the absence of water for 9 days (≥90%, 5) as visualized by a general thicker, green canopy. **D**) Fluid usage of 5 week old NS or S (SaltSym, TempSym1, and TempSym2) rice plants (N = 60) as ml of fluid used over a ten day period. Statistical analysis (Duncan's multiple-range test) indicated significant differences in fluid usage (SD≤7.51; P<0.05) with all three symbionts using less fluid compared to NS plants.

**Figure 4:**
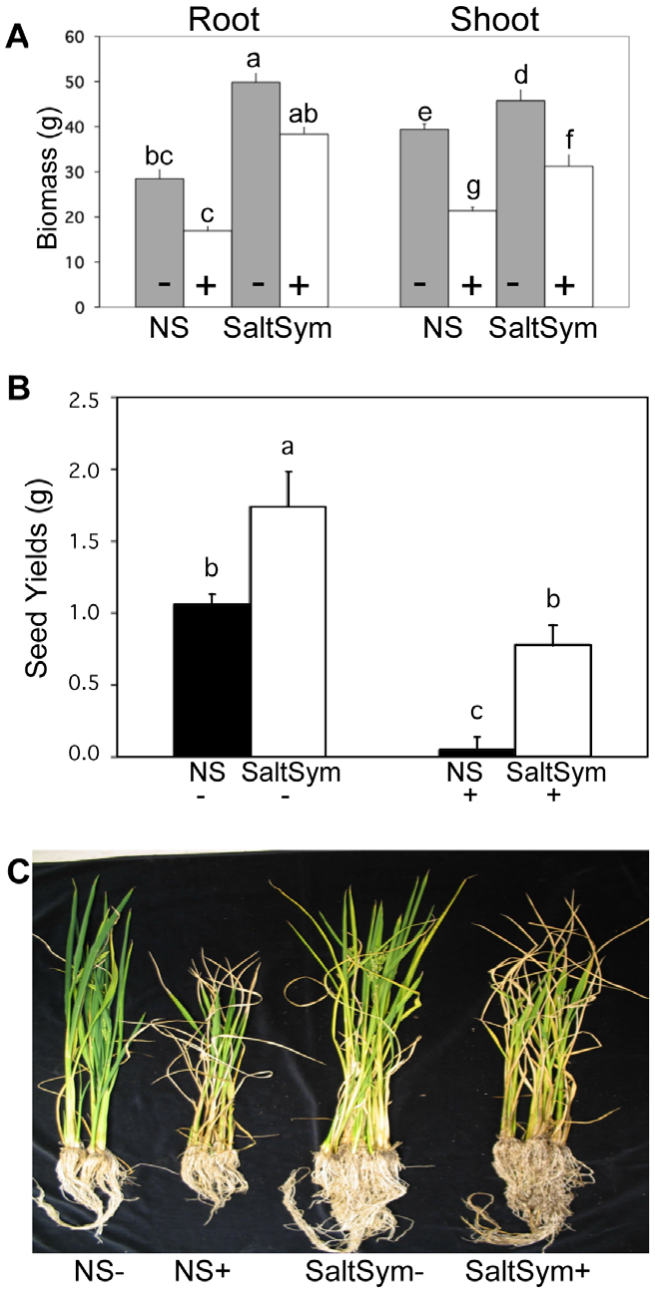
The effect of stress on root, shoot and seed yields of mature plants under greenhouse conditions. Two month old NS and S (SaltSym) rice plants (N = 30) were +/− exposed to a gradual increase in NaCl concentrations (0–300 mM) over an additional three-month period. Non-stressed NS and S (SaltSym) plants were hydrated with 1x Hoagland's solution supplemented with 5 mM CaCl_2_ throughout the length of the study (5 months total). Stress treatments began after 2 months and plants were exposed to 100 mM NaCl for 3 weeks, then increased 200 mM NaCl for an additional 3 weeks, and then increased and maintained at 300 mM NaCl until the completion of the study (5 months total). **A**) Root and shoot biomass (N = 5) of NS and S (SaltSym) plants in the absence (-; grey bar graphs) and presence (+; white bar graphs) of salt stress. Statistical analysis (Duncan's multiple-test range) indicated S plants had significantly larger shoot biomass in the presence and absence of stress (SE<2.88; P<0.0001). Significant differences between S and NS treatments in root size were observed (SE<2.81; P<0.0031) in +/− salt stress treatments. No significant differences in root biomass were observed when comparing NS+ versus NS-, or S+ versus S- (Values with the same letters are not significantly different). **B**) Seed yields of plants (N = 25) of NS and S (SaltSym) plants in the absence (-) and presence (+) of salt stress. Statistical analysis (Duncan's multiple-test range) indicated S+ plants had higher yields than NS+ plants, and S- plants had higher yields than NS- plants (SD≤0.17;P<0.01). **C**) Representative photo of NS and S (SaltSym) plants grown in the absence (-) and presence (+) of a gradual increase in salt stress (0–300 mM NaCl, see above).

Symbiotically induced seedling developmental rates (Fig. 3) and young plant growth increases were dramatic in both roots and shoots of five-week old plants (Fig. 4). Differences in growth and root development of S and NS plants were observed using time-lapse photography under 14 hr light cycles (Fig. 2). Rice seedlings were exposed for 48 hr to water (NS) and fungal spores (S) at which point germination occurred (t = 0, Fig. 2). Germinated seedlings were than embedded in the silica sand layer on top of the time-lapse apparatus (see Materials and Methods). NS plant shoot growth began before root growth initiated while S plants increased root mass prior to substantial shoot growth. By the time S plants began developing root hairs (day 6), NS plants had not yet begun significant root growth.

Mycelia of endophytes grown in liquid culture (SaltSym & TemSym1) were assayed for the production of IAA and IAA-like plant growth stimulating compounds. The colorimetric assay detects indole compounds, some of which are important in promoting plant growth [41]. This assay was specific enough to detect Indoles and not tryptophan. Endophytes were grown in liquid media for 21 days (Table 2). Endophytes grown in Mathur's Media with the addition of Trp produced 200–500 ppm IAA within 5 days, and levels were maintained until the last time point taken after 21 days. In the absence of Trp, or growth in other than Mathur's Media (such as PDA), no IAA was detected. These results suggest that the growth response in symbiotic plants may be due to endophyte production of IAA or IAA like plant growth stimulating compounds, which can be induced in the presence of Trp, and possibly suppressed by media components (e.g. sucrose or other amino acids).

**Table 2:** IAA production of endophytes in media and *in planta.*

Isolate^a^	Media^b^	IAA (ppm)±SD^c^
SaltSym	MS	ND
	MS+Trp	500+40
	PDA	ND
	PDA+Trp	ND
TempSym1	MS	ND
	MS+Trp	200±35
	PDA	ND
	PDA+Trp	ND

Isolate		IAA (ppm)^c^
NS	***in planta*** d	ND
SaltSym	***in planta*** d	ND
TempSym1	***in planta*** d	ND

a Fungal endophyte isolates tested for production of IAA.

b Endophytes were grown in liquid media in the presence (+) or absence of 0.1% tryptophan (Trp) for 21 days.

c Indole-3-acedtic acid (IAA) detected in parts per million (ppm).

ND = not detected.

d Ten, five day old seedlings nonsymbiotic (NS) or symbiotic with SaltSym or TempSym1were assessed for IAA.

Additional analysis revealed that IAA was not detected in five-day old S and NS rice seedlings irrespective of a significant growth response in S plants (Table 2). The absence of detectible levels of IAA in symbiotic rice seedlings may reflect levels of IAA below detectable range for the assay, the physiological status of plants at the time of analysis, or lack of sufficient Trp for fungal biosynthesis. Regardless, these results suggest that the potential role of endophytes and IAA production *in planta* needs to be addressed in greater detail.

Growth and biomass differences under greenhouse conditions translated into yield differences with NS plants producing less than 10% of the seed yield of SaltSym plants in the presence of salt stress (Fig. 4B). A seedling development assay indicated that germinated seeds of S and NS plants had equivalent levels of viability and development at temperatures>15°C (≥ 95%; Figure 3B).

### Stress Tolerance (cold, salt and drought)

The ability of habitat-adapted endophytes to confer significant levels of stress tolerance to young rice plants was assessed in double-decker Magenta boxes [33]under laboratory conditions. As anticipated, SaltSym conferred significant levels of salt tolerance to rice allowing them to grow at 300 mM NaCl (Fig. 3). Rice plants were grown for five weeks without stress before continual exposure to NaCl for three weeks.

To determine the impact of salt stress on mature plants (plants taken to seed set), SaltSym and NS plants were grown under greenhouse conditions, without salt stress for two months, and then gradually exposing plants to increasing levels of salt from 100 mM–300 mM NaCl prior to seed production. Seed production was measured after 5 months of growth, of which, approximately 6 weeks of that time frame, involved exposure to the highest level of salt concentrations of 300 mM NaCl. During the course of these studies, rice plants continued to grow even when exposed to high levels of salt, resulting in little root biomass differences within a treatment, but significant differences in shoot biomass that was +/− exposed to salt stress (Fig. 4). Analysis of roots and shoot showed an increase in the root biomass of S plants in the presence and absence of salt stress, and shoot biomass of S plants in the presence and absence of stress, compared to NS plants. These results suggest that through symbiosis, endophytes may play a dual role in growth enhancement and salt stress tolerance. When yields were assessed, a significant difference in seed production was observed in S plants in the presence and absence of stress when compared to NS plants (Fig.4). Analysis of % biomass change in NS+ samples showed a decrease in root (30.84%) and shoot (26.13%) tissues, respectively, when exposed to salt stress compared to the same plant treatment (root and shoot) in the absence (NS-) of stress. Symbiotic (SaltSym+) plants however, showed a statistically lower %biomass change in root (15.04%) and shoot (19.54%) tissues, respectively, in salt stressed plants compared to the same plant (root and shoot) treatment in the absence (SaltSym-) of stress. Although seed production by S plants was decreased by salt stress, the levels were not significantly different from NS plants grown without stress.

TempSym1 was isolated from plants growing in geothermal soils that achieve temperatures of up to 60°C during dry summers and around 20°C in the winter [29], and TempSym2 isolated from the same plant species in cooler geothermal soils (<30°C in summer & 5°C in winter). Although TempSym1 is adapted to high soil temperatures, the canopy of the plants in which both endophytes were originally isolated, experience below freezing temperatures in the winter and continue to produce vegetative tissues. Therefore, TempSym1 & 2 (both *C. protuberata* isolates) were assessed for the ability to confer cold tolerance to rice seedling development (Fig. 3). This was analyzed by assessing the seedling development of inoculated and non-inoculated treatments incubated at temperatures below 20°C. To ideally make observations concerning the impacts of cold stress on seedling shoot and root development, rice seeds were surface sterilized and imbibed (see Materials and Methods: plant colonization section), seeds carefully selected that showed a small white tissue protuberance indicating a high potential success rate of germination (unpublished observations made by authors Redman & Kim). Samples were then inoculated or non-inoculated and placed at 5–20°C. The % seedling development (appearance of roots and shoots) was assessed after ten days. Symbiotic seedlings had development frequencies of greater than 90% at all of the temperatures tested, while uninoculated seeds achieved greater than 90% only at 20°C, and a substantial decrease of 70% and 45%, at 10° and 5°C, respectively.

All three endophytes (TempSym1 and 2, and SaltSym) were tested for the degree of drought tolerance they could confer in the absence of any other stress. This was done by determining time-to-wilt after the cessation of watering (Fig. 3). NS plants wilted after 3 days while TempSym1 inoculated plants did not wilt for 9 days. The other two endophytes in this study (TempSym2 and SaltSym) also conferred drought tolerance (7 and 9 day delay in wilt, respectively, not shown) compared to NS plants. Previous studies indicated that these endophytes decrease plant water consumption, which may explain symbiotically conferred drought tolerance [33]. A comparison of water consumption in S and NS rice plants revealed significant decreases (20–30%) by S plants with TempSym1 inoculated plants using the least amount of water (Fig. 3).

### Physiological responses (osmolytes, ROS)

**Figure 5:**
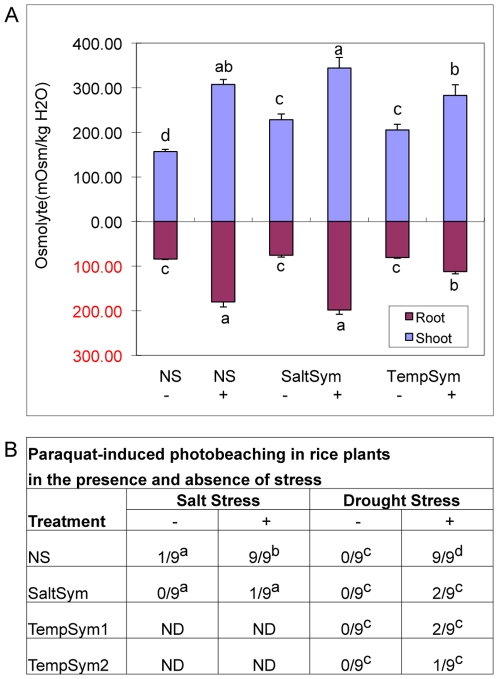
Effect of symbiosis on plant osmolyte concentrations and paraquat-induced photobleaching (ROS) under laboratory conditions. **A**) Five week old rice plants (N = 30) that were NS or colonized with SaltSym or TempSym1 exposed for ten days in the absence (-) and presence (+) of salt stress (300 mM NaCl), at which point, the effects of stress began to show in NS plants treatments (≥70% wilted +/−chlorosis). SaltSym imparts salt tolerance and TempSym1 does not. Osmolyte concentrations (milliosmoles per kg wet weight) of roots and shoots were assessed and statistical analysis (Duncan's multiple-range test; SE≤9.98 &<23.73 for root, and shoot, respectively; P<0.0001 for root and shoot) indicated significantly higher levels in the shoots of S plants compared to NS plants in the absence of salt stress, and no statistical differences between treatments in the presence of salt stress. No significant differences were observed in roots in the absence of salt stress. In the presence of stress, Tempsym1+ showed significantly lower level of osmolytes than SaltSym+ and NS+ treatments. Values with the same letters are not significantly different. **B**) NS and S (SaltSym and TempSym1 & 2) plants exposed to salt (300 mM NaCl, 10 days) and drought stress (3 days) were tested for paraquat-induced photobleaching (ROS activity). Time points were chosen when symptoms began to appear (wilting and chlorosis) in NS stressed plants. Leaf disks (N = 9) from 9 independent plants were used for ROS assays. Leaf disks were sampled from leaf tissues of similar size, developmental age, and location for optimal side-by-side comparisons. Values indicate the number of leaf disks out of a total of nine that bleached white after exposure to paraquat indicating ROS generation. Statistical analysis (Duncan's multiple-range test) indicated that in the absence of stress, little to no (0–11%) photo beaching occurred in all the treatments. In contrast, significant differences occurred with 100% of the NS plant disks for both salt and drought stress bleaching white compared to only 11–22% of the S plant disks (P<0.0001). ND =  not determined.

Two common plant responses to abiotic stresses such as heat, salt and drought involve regulating the levels of osmolytes and reactive oxygen species (ROS) [12]. Therefore, we analyzed plant osmolyte concentrations and sensitivity to ROS before and after exposure to salt and drought stress (Fig. 5). In the absence of salt stress, S plants had higher levels of osmolytes in shoots compared to NS plants but equivalent levels in the roots. In the presence of salt, all of the plants increased osmolyte levels in both shoots and roots, regardless of the treatment, with SaltSym expressing the highest osmolyte levels.

Excised leaf discs from plants grown in the absence of stress or exposed to salt and drought stress were analyzed for ROS sensitivity. One way to mimic endogenous production and assess sensitivity to ROS is to expose photosynthetic tissue to the herbicide paraquat, which is reduced by electron transfer from plant photosystem I and oxidized by molecular oxygen resulting in the generation of superoxide ions and subsequent photobleaching [42]. Leaf discs from plants that were not exposed to stress remained green indicating that ROS was not produced by exposure to paraquat (Fig. 5). When exposed to salt stress, plants colonized with SaltSym showed no significant photobleaching (11%) while NS plant tissues bleached white (100%). Similarly, S plant tissues exposed to drought stress showed no significant photobleaching (11–22%) while NS plants did (100%).

## Discussion

Rice plants were adapted to cold, salt and drought stress simply by colonization with Class 2 fungal endophytes. Salt and temperature stress tolerance are habitat-adapted traits of the endophytes evaluated in this study [33]. SaltSym, derived from coastal plants (*Leymus mollis)* exposed to high salt stress confer salt tolerance, and not temperature tolerance. TempSym1 & 2 were isolated from *Dichanthelium lanuginosum* thriving in geothermal soils conferred temperature tolerance and not salt tolerance. Since TempSym1 & 2 originated in geothermal soils differing in summer maximum and winter minimum temperatures, we anticipated that TempSym2 and not TempSym1 could confer cold tolerance. The fact that both endophytes conferred cold tolerance may reflect the cold winter temperatures plants experience above ground rather than in the soil.

Initial cell signaling and biochemical pathways involved in both hot and cold temperature stress responses begin with the same root physiological processes and later branch off into unique pathways [12]. It is tempting to speculate that TempSym1 & 2 regulate early events (as our ROS studies indicate) in the plant temperature response such that downstream responses do not occur resulting in tolerance to heat and cold.

To make observations concerning the impacts of cold stress on seedling shoot and root development, rice seeds showing a small white tissue protuberance (indicating a high potential success rate of germination) were used for the cold stress assays. Cold stress tolerance was conferred to germinated seeds under laboratory conditions by TempSym1 less than 24 hr post inoculation of seeds resulting in greater than 90% seedling development at temperatures between 5°–20°C (Fig.3). It is possible that TempSym1 either allows plants to increase metabolic rates at low temperatures or increase metabolic efficiency to overcome affects of low temperature. Symbiotically induced metabolic efficiency was also observed in laboratory studies showing decreased water consumption and increased biomass in S plants.

Salt and drought stress were tested under greenhouse and growth chamber conditions. SaltSym conferred salt tolerance, allowing plants to grow when continually exposed to a solution of 300 mM NaCl (Fig. 3). More importantly, a gradual increase in salt exposure of mature plants effectively eliminated seed production in NS plants. Although mature S plants had reduced seed production under salt stress compared to non-stressed plants, salt stressed S plants produced similar amounts of seed as NS plants grown in the absence of salt stress (Fig. 4). The number of stems of S plants was statistically higher (data not shown P≥0.05)) than NS plants which would prove to be beneficial to overall plant health and yields under field conditions. The levels of salt used in these studies are similar to those occurring in agricultural lands after tsunamis or tidal surges [37]. Therefore, we anticipate that using SaltSym may allow growers to mitigate the impacts of salt inundation.

A common physiological response to salt stress is an increase in the production of osmolytes [12]. In the absence of salt stress, osmolyte levels were similar in the roots of S and NS plants but significantly higher in the shoots of S plants compared to NS plants (Fig. 5). Upon exposure to salt stress both S and NS plants significantly increased osmolyte levels, but their responses differed with NS plants increasing by approximately 50% and symbiotic plants increasing approximately 30% compared to non-stressed plants. This was unexpected as previous results, albeit with a different stress, indicated that in the presence of heat stress, osmolytes increased significantly in nonsymbiotic plants but either increased slightly or not at all in S plants [33]. These results suggest that osmolyte production in symbiotic plants varies either with the type of stress, endophyte genotype, and/or plant genotype. Regardless, since all plant treatments in this study responded in a similar manner (an overall increase in the presence of stress regardless of the treatment), it appears that osmolyte production alone is not responsible for symbiotic adaptation of rice plants to salt stress.

All three endophytes conferred drought tolerance to rice plants delaying wilt, 2–3 times beyond that of NS plants (Fig. 3). Although the mechanisms of endophyte conferred drought tolerance are not known, delayed wilt time correlated with a reduction of water usage (20–33%). Production of ROS is correlated with the early events in the plants stress response system. Our studies indicated that drought and salt tolerance in S plants correlated to decreased ROS activity (Fig. 5). All of the NS leaf tissues photobleached in the presence of paraquat while only 0–22% of the S plants showed any photobleaching. Increases in ROS are common to all stresses as a result of stress-induced metabolic imbalances [42], [43]. The data suggest that in the presence of stress, either rice plants remain metabolically balanced or over express ROS scavenging antioxidation systems. Nevertheless, decreased ROS activity in S plants correlates strongly with stress tolerance and may play a critical role in the process.

The endophytes increased the potential fitness of rice plants by enhancing growth, development, biomass and yield in the presence and absence of stress as observed under laboratory and greenhouse conditions. The influence of the endophytes on plant growth and development was significant. Assessing percent biomass changes in the treatments revealed that there was a 6.59% decrease in shoot biomass in NS compared to SaltSym plants exposed to stress. In roots, the effects were more dramatic with 15.8% root biomass decrease in NS plant compared to SaltSym plants exposed to stress. These results suggest that SaltSym plants are able to better deal with the negative effects of salt stress than their NS counterparts. Although the basis of endophyte induced growth promotion is not known, the fungal endophytes are able to produce significant amounts (≤500 ppm) of IAA, a plant growth hormone, when grown in culture (Table 2). Remarkably, enhanced growth was observed within 24 hr of colonization and observations indicated endophytes influenced the allocation of resources into roots and shoots (Fig. 2). Time-lapse imaging revealed that nonsymbiotic plants preferentially allocated resources into shoots prior to substantial root growth while S plants increased root mass prior to shoot growth. This is in contrast to plants grown under continual light: NS plants equally distributed resources into roots and shoots while the shoots of S plants had limited growth until root hairs were developed [44]. The difference in resource allocation in plants grown under continual light and a 14 hr light cycle indicates that under light conditions (12–14 hr) occurring during crop production, the impacts of symbiosis are much greater than under continual light. It is tempting to speculate that since symbiotic plants use less water, produce greater biomass, and have higher yields; the endophytes may allow rice plants to achieve greater metabolic efficiency.

Taxonomically, *L. mollis*, *D. lanuginosum* and rice are in the family (Poaceae) but are in different subfamilies (Pooideae, Panicoideae and Ehrhartoideae, respectively). Previous studies indicated that SaltSym and TempSym1 could also colonize and confer habitat-specific stress tolerances to the eudicot tomato [27], [33] suggesting that the symbiotic communication responsible for stress tolerance is conserved among plant lineages. The ability of endophytes to colonize and confer stress tolerance, increase yields and biomass, and disease resistance [28] to genetically unrelated plant species suggests that they may be useful in adapting plants to drought, salt and temperature stresses that are predicted to worsen in future years due to climate change.

## Materials and Methods

### Assessing endophyte colonization from plant tissues

At the beginning and end of each experiment, the efficiency of endophyte colonization of inoculated plants and the absence of endophytes in mock inoculated controls was assessed as follows: a subset of at least 10% of laboratory and greenhouse plants were assessed for colonization. Plants were washed until soil debris was removed, placed in to plastic zip-loc baggies and surface sterilized as previously described [28], [45]. Using aseptic technique, plants were cut into sections representing the roots and stem sections, imprinted [46], plated on fungal growth media (see below), and incubated 5–7 days at 22°C with a 12 hr light cycle (cool fluorescent lights) to allow for the emergence of fungi. Upon emergence, fungal endophytes were identified using microbiological and molecular techniques as previously described [33]. The effectiveness of surface sterilization was verified using the imprint technique [46].

#### Fungal cultures

*Fusarium culmorum* and *Curvularia protuberata* species (Table 1) were cultured on 0.1X potato dextrose agar (PDA) medium supplemented with 50–100 µg/ml of ampicillin, tetracycline, and streptomycin, and fungal cultures grown at 22°C with a 12 hr light regime. After 5–14 days of growth, conidia were harvested from plates by adding 10 ml of sterile water and gently scraping off spores with a sterile glass slide. The final volume of spores was adjusted to 100 ml with sterile water, filtered through four layers of sterile cotton cheesecloth gauze and spore concentration adjusted to 10^3^–10^4^ spores/ml.

#### Rice Varieties

*Oryza sativa*, var. M-206 (subspecies Japonica) and Dongjin (subspecies Indica) were collectively used in greenhouse and laboratory studies. M-206 is a variety predominantly grown in Northern California and Dongjin in South Korea.

#### Plant colonization

For laboratory and greenhouse studies, seeds were surface sterilized in 2.5% (v/v) sodium hypochlorite for 24–48 hr, rinsed with 5–10 volumes of sterile distilled water, and imbibed in 1–2 volumes of water for 8–12 hr. Seeds were germinated on 1% agar water medium, maintained at 26°–30°C and exposed to a 12 hr fluorescent light-regime. To ensure that our studies began only with nonsymbiotic plants, seedlings that showed no outgrowth of fungi into the surrounding media were chosen and transplanted. Any seedlings showing outgrowth of fungi, were discarded. Endophyte-free plants (up to 20 plants/magenta box depending upon the study) were planted into sterile double-decker magenta boxes (modified magenta boxes to hold soil or sand in upper chamber, and fluid in lower chamber that is wicked-up through a cotton rope; [33]) containing equivalent amounts (380+/−5 g) of sterile-sand, or Sunshine Mix #4 [Steuber Distributing Co., WA, USA (40+/−0.5 g)]. The lower chamber was filled with 200 ml of sterile water or 1x Hoagland's solution supplemented with 5 mM CaCl_2_. After 1 week, plants were either mock inoculated with water (nonsymbiotic) or inoculated with fungal endophytes by pipetting 10–100 ul of spores (10^3^–10^4^/ml) at the base of the crowns or stems. Plants were grown under a 12 hr light regime at 26°–30°C for 3–5 weeks for laboratory, and 2 months for greenhouse studies prior to imposing stress.

#### Growth response and development

Symbiotically induced growth response of root and shoot development was visualized in seedlings through time-lapse photography. Nonsymbiotic (NS) and symbiotic (S) seeds were generated by placing surface sterilized seeds on agar plates (containing 1x Hoagland's solution supplemented with 5 mM CaCl_2_) and flooding the plates with water (mock-inoculated) or fungal spores (10^2^–10^3^/ml) for 48 hr. Three geminated seedlings were chosen at random from a total of N = 100 seeds. The representative seedlings were placed onto a vertical plant growth apparatus for photographic monitoring. The apparatus was comprised of two 3 mm thick glass plates (30 cm×30 cm), with a divider (high pressure tubing) placed down the center, and the whole apparatus sealed around the edges using Tygon tubing and clamps to generate two separated leak-free compartments. One hundred fifty ml of 1x Hoagland's media (supplemented with 5 mM CaCl_2_ and 1% agarose) was poured into each compartment and allowed to solidify. A thin layer (2–3 mm) of silica sand was placed on top of the solid matrix. All components and media used were either surface sterilized in 70% EtOH or autoclaved prior to assembly. Three germinated seedlings were then embedded in the sand on each side of the compartment. Seedlings were maintained at 26°C in a 14 hour light/dark regime using daylight balanced fluorescent studio lights. Photos were taken every 20 min using a Canon PowerShot G5 camera and a Pclix infrared Controller (Toronto, Canada).

#### Abiotic stresses

Greenhouse and laboratory experiments were performed with plants grown in magenta boxes at 26°–30°C in a temperature controlled room with a 12 hr fluorescent light regime. Magenta boxes were randomly placed in different locations on shelves in the growth room for salt and drought stress experiments. Each experiment was repeated three times and the images in the figures are representative of all replications of each treatment.

Magenta boxes contained<20 plants and the total number of plants/replication is indicated as (N = XX) in the figure legends. The health of plants was assessed on a scale of 1–5 (1 = dead, 2 = severely wilted and chlorotic, 3 = wilted +/− chlorosis, 4 = slightly wilted, 5 = healthy w/o lesions or wilting), and is listed in the figure legends.

Control plants – All control plants were maintained at 26–30°C and hydrated throughout the experiment with sterile water or 1x Hoagland's solution supplemented with 5 mM CaCl_2_.

Salt – plants were exposed to 100–300 mM NaCl in 1x Hoagland's solution supplemented with 5 mM CaCl_2_ (referred to as 100 mM, 200 mM and 300 mM NaCl solution) for 10–21 days for laboratory studies by filling the lower chamber of the double-decker magenta boxes with 200 ml of the salt solutions. Greenhouse studies with mature plants were exposed to 100 mM–300 mM NaCl for up to 3 months (see below). After plants were showing symptoms (i.e., nonsymbiotic plants dead or severely wilted), they were re-hydrated in sterile water devoid of NaCl for 48 hr, plant health assessed and photographed. All laboratory assays were repeated three times.

Drought – watering was terminated for 3–15 days by decanting off the fluid in the lower chamber of the double-decker magenta box and letting the plant soils dry out over-time. A hydrometer (Stevens Vitel Inc.) was used to ensure that soil moisture levels were equivalent between treatments when watering was terminated. After plants showed symptoms (i.e., NS plants dead or severely wilted), they were re-hydrated in sterile water for 48 hr, plant health assessed and photographed. All assays were repeated three times.

#### Plant water usage and biomass

Water consumption was measured in double-decker magenta boxes. Initially, 200 ml of 1x Hoagland's solution supplemented with 5 mM CaCl_2_ were placed in the lower chamber. Fluid remaining in the lower chamber after 10 days of plant growth was measured, and water usage calculated as ml consumed/10 days. All assays were repeated three times.

#### Cold Stress

The effect of cold stress was assessed using surface sterilized and imbibed seeds (see plant colonization above for details) that were either mock-water or fungal spore (10^2^–10^3^/ml) inoculated by immersion in solutions for 48 hr with gentle agitation. Twenty NS and S seedlings (seeds exhibiting a small white tissue radical - indicative of germination) were placed on solid water agar (1%) plates and incubated at 5°C, 10°C, 15°C, and 20°C, and the % seedling development assessed after 10 days. All assays were repeated three times.

#### Salt stress versus plant biomass and yields

The effect of symbiosis on the growth and yield of mature rice plants exposed to prolonged salt stress (3 months) was tested with NS and S (endophyte conferring salt tolerance referred to as SaltSym)] plants under greenhouse conditions. Plants were generated using standard protocols (as described above). Two-month old plants were either maintained as control non-stressed plants and watered with 1x Hoagland's solution supplemented with 5 mM CaCl_2_ or salt stressed plants exposed to 100 mM–300 mM NaCl in 1x Hoagland's solution supplemented with 5 mM CaCl_2_. Stressed plants were initially exposed to 100 mM NaCl solution for 3 weeks, exposed to 200 mM NaCl solution for an additional 3 weeks, and then exposed to 300 mM NaCl solution until the termination of the studies (approximately 6 weeks for a total of 5 months for the study). Upon termination of the studies, the spikes were removed and weights determined (g). Prior to plant biomass assessment (wet weight), roots and shoots were gently washed to remove dirt and debris. Roots and stems were than blotted dry, cut into root and shoot sections, and weights determined (g). Representative photos were taken of all treatments.

#### Colony Forming Units (CFU)

NS and S plants were surface sterilized (described above) and 5 plants (total of 0.5 g) pooled to obtain equal amounts of roots and lower stems. Plant tissues were homogenized (Tekmar tissue homogenizer) in 10 ml of STC osmotic buffer (1M Sorbitol, 10 mM TRIS-HCl, 50 mM CaCl_2_, pH 7.5) on ice and 100 µl plated onto 0.1XPDA fungal growth medium (see above). After 5–7 days at 25°C, CFU were assessed. All assays were repeated three times.

#### Plant osmolyte concentrations

NS and S plants exposed to +/− salt stress were analyzed for osmolyte concentrations. Equivalent amounts of root and lower stem tissues (100 mg total) from 3–5 plants/condition were ground in 500 ul water with 3 mg sterile sand, boiled for 30 min, samples cooled to 25°C, centrifuged for 5 min at 6 K rpm, and osmolytes measured with a Micro Osmometer 3300 (Advanced Instruments). All assays were repeated three times.

#### Reactive Oxygen Species (ROS)

NS and S plants were exposed to +/− salt (300 mM NaCl solution) and drought stress for 3–10 days and leaf tissue samples taken just prior to or when slight to moderate stress induced symptoms were observed. Using a cork borer, leaf discs (2 mm) were obtained from each of 3 replicate plants from different magenta boxes and placed on a solution of 1 uM of the herbicide paraquat (N,N'-Dimethyl–4,4′-bipyridinium dichloride, Syngenta, Greensboro, NC) and incubated at 22°C under fluorescent lights. After 48 hr exposure to paraquat, leaf discs were photographed to document chlorophyll oxidation visualized by tissue bleaching. All assays were repeated three times.

#### IAA Assays

*Fusarium* sp. and *Curvularia* sp. were cultured on 0.02-0.1X potato dextrose agar (PDA) medium and 0.2-1X modified Mathur's media [MS [47]]. Both media were supplemented with 50–100 ug/ml of ampicillin, tetracycline, and streptomycin. Indole-3-acetic acid (IAA) assays of mycelia grown in liquid media was assessed by growth on 0.1X PDA or 1X MS media as follows: 3–5 plugs of fungal mycelia plugs were inoculated into liquid media and grown for 2 days at 22°C with agitation (220 rpm). After 2 days, mycelia were blended (Tekmar) and grown an additional 1–2 days until a dense suspension of mycelia was achieved. Mycelia were filter through 4 layers of sterile gauze and washed with 10 volumes of sterile water. Ten percent mycelia (g/100 ml) was inoculated into 0.2X MS liquid media +/−0.1% tryptophan (Trp) and grown with agitation (220 rpm) for up to 3 weeks. One ml of supernatant was collected at various time points (24 hr–21 days), the supernatant passed through spin columns (Millipore centrifugal filter units) to remove mycelia and pigment, and IAA assessed using Fe-HClO_4_ solution colorimetric assay (OD = 530 nm) protocol [41] and levels compared to an IAA (Sigma) standard curve of 0–1000 ppm. IAA levels in NS and S [*F. culmorum* isolate FcRed1 (SaltSym) and *C. protuberata* isolate Cp4666D (TempSym1)] 5 day old rice seedlings were assessed by grinding x10 plants (seeds removed) in liquid N_2_, re-suspending plant debris in an equal volume of sterile water, vortexed 1 min, incubated for 30 min at 22°C, and the supernatant passed through spin columns and assessed for IAA as described above. IAA standards were passed through spin columns with no hindrance. All assays were conducted three times.

#### Statistical analysis

P values were determined by Duncan's multiple-range test and data analyzed using SAS [48].
